# Dietary grape seed proanthocyanidins inactivate regulatory T cells by promoting NER-dependent DNA repair in dendritic cells in UVB-exposed skin

**DOI:** 10.18632/oncotarget.17867

**Published:** 2017-05-15

**Authors:** Mudit Vaid, Ram Prasad, Tripti Singh, Santosh K. Katiyar

**Affiliations:** ^1^ Birmingham Veterans Affairs Medical Center, Birmingham, AL, USA; ^2^ Department of Dermatology, University of Alabama at Birmingham, Birmingham, AL, USA

**Keywords:** grape proanthocyanidins, ultraviolet radiation, photocarcinogenesis, contact hypersensitivity, regulatory T cells

## Abstract

Ultraviolet B (UVB) radiation induces regulatory T cells (Treg cells) and depletion of these Treg cells alleviates immunosuppression and inhibits photocarcinogenesis in mice. Here, we determined the effects of dietary grape seed proanthocyanidins (GSPs) on the development and activity of UVB-induced Treg cells. C3H/HeN mice fed a GSPs (0.5%, w/w)-supplemented or control diet were exposed to UVB (150 mJ/cm^2^) radiation, sensitized to 2,4-dinitrofluorobenzene (DNFB) and sacrificed 5 days later. FACS analysis indicated that dietary GSPs decrease the numbers of UVB-induced Treg cells. ELISA analysis of cultured sorted Treg cells indicated that secretion of immunosuppressive cytokines (interleukin-10, TGF-β) was significantly lower in Treg cells from GSPs-fed mice. Dietary GSPs also enhanced the ability of Treg cells from wild-type mice to stimulate production of IFNγ by T cells. These effects of dietary GSPs on Treg cell function were not found in *XPA*-deficient mice, which are incapable of repairing UVB-induced DNA damage. Adoptive transfer experiments revealed that naïve recipients that received Treg cells from GSPs-fed UVB-irradiated wild-type donors that had been sensitized to DNFB exhibited a significantly higher contact hypersensitivity (CHS) response to DNFB than mice that received Treg cells from UVB-exposed mice fed the control diet. There was no significant difference in the CHS response between mice that received Treg cells from UVB-irradiated *XPA*-deficient donors fed GSPs or the control diet. Furthermore, dietary GSPs significantly inhibited UVB-induced skin tumor development in wild-type mice but not in *XPA*-deficient mice. These results suggest that GSPs inactivate Treg cells by promoting DNA repair in dendritic cells in UVB-exposed skin.

## INTRODUCTION

It is well established that excessive exposure of the skin to solar ultraviolet (UV) radiation results in suppression of the immune system. This UV-induced immunosuppression has been implicated in the UV-induced development of skin tumors. In humans, chronically immunosuppressed patients who live in regions of intense sun exposure have an exceptionally high rate of non-melanoma skin cancer [1–4]. The association between immunosuppression and development of skin tumors also is suggested by the high incidence of skin cancers, especially squamous cell carcinomas (SCCs), among organ transplant recipients who require prolonged immunosuppressive therapy [5–8]. A clear association between UV-induced development of skin tumors and UV-induced immunosuppression has been demonstrated in mice [1–3]. Several lines of evidence indicated that UV-induced T suppressor cells or regulatory T cells (Treg) play a central role in UV-induced immunosuppression and initiation of skin carcinogenesis [9, 10] and that depletion of UV-induced suppressor T cells can inhibit UV-induced skin carcinogenesis [11]. Elmets *et al*. assessed UVB-induced immunosuppression by analysis of the effects of the UVB irradiation on the contact hypersensitivity (CHS) response, which is considered a prototypic T cell-mediated response [12]. Using this model, they demonstrated that UVB exposure results in the emergence of specific suppressor T cells and that these cells may be responsible for the development of immune-tolerance against the sensitizing hapten. Subsequent characterization of these suppressor T cells indicated that they express CD4 and CD25 [13] as well as the negative regulatory molecule CTLA-4 (CD152) and are therefore now classified as regulatory T cells (Treg cells) [14].

A molecular mechanism that has been shown to link UVB exposure to immunosuppression and initiation of photocarcinogenesis in mice is UV-induced DNA damage, particularly in the form of cyclobutane pyrimidine dimers (CPD), in the antigen presenting cells of the skin [15, 16]. Repair of CPDs in epidermal Langerhans cells, whether by topical application of exogenous DNA repair enzymes [16] or by injection of the immunostimulatory cytokine IL-12 [17, 18], which has the ability to repair UV-induced DNA damage, has been correlated with inhibition of UV-induced immunosuppression. The UVB-induced DNA damage in the Langerhans cells compromises the ability of these skin antigen presenting cells (APCs) to present antigen to T cells and contributes to the generation of the immunosuppressive Treg cells. Collectively, these data indicate that Treg cells induced by defective antigen presentation by UVB-damaged skin APCs are key mediators of UV-induced immunosuppression.

To develop more effective and mechanism-based strategies for the chemoprevention of skin cancer, we are assessing the effects of selected phytochemicals, including grape seed proanthocyanidins (GSPs), on UV-induced immunosuppression using preclinical animal models. GSPs consist of dimers, trimers, tetramers and oligomers of monomeric catechins or epicatechins [19–21] and possess anti-oxidant and anti-inflammatory activities [22–25]. We have shown previously that provision of a GSPs-supplemented diet inhibits UV-induced skin tumor development in mice as assessed by analysis of tumor incidence and tumor multiplicity [25]. Dietary GSPs also inhibit UVB-induced immunosuppression in the mice, and that is associated with both an increase in the levels of the immunostimulatory cytokine IL-12 and enhancement of DNA repair activity in the UVB-exposed skin [26]. We have further found that dietary GSPs inhibit UVB-induced immunosuppression in the CHS mouse model, at least in part, through their ability to restore the functional activity of UVB-irradiated dendritic cells (DCs) [27]. However, there is only limited information regarding the effects of dietary GSPs on the development and/or function of Treg cells. Moreover, it is not known if there is any association between GSPs-induced stimulation of DNA repair and its effects on the development of Treg cells in UVB-exposed animals. We therefore tested whether dietary GSPs inhibit UVB-induced immunosuppression by affecting the numbers or functional activity of Treg cells. To determine the association of the effects of dietary GSPs on Treg cells and the ability of dietary GSPs to repair damaged DNA in UV-exposed mouse skin, we used xeroderma pigmentosum complementation group-A (*XPA*) deficient mice, which are incapable of repairing UVB-induced DNA damage through the nucleotide excision repair (NER) mechanism.

## RESULTS

### Dietary GSPs inhibit the development of UVB-induced Treg cells and decrease the functional activation of UVB-induced Treg cells that express CD4/CD25/Foxp3

**Figure 1:**
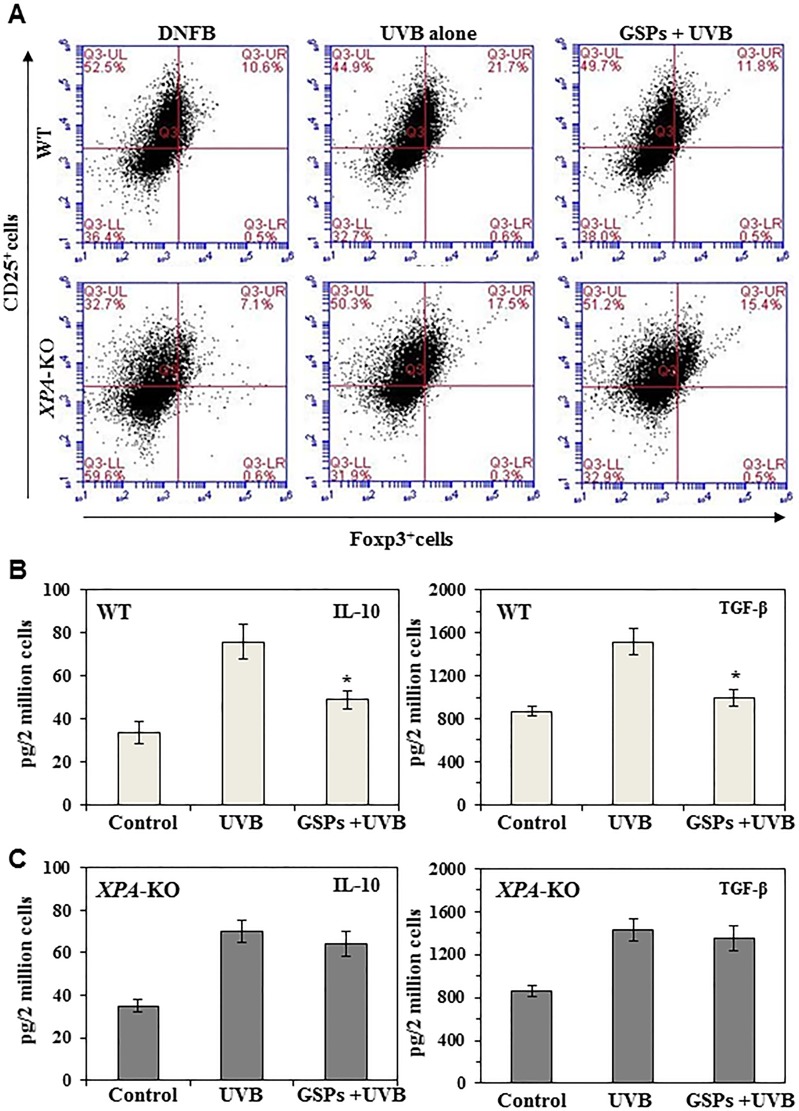
Effect of dietary GSPs on the levels of immunosuppressive cell population and their cytokine secretion Dietary intake of GSPs affects the numbers of Treg cells (CD25^+^/Foxp3^+^) and inhibits secretion of immunosuppressive cytokines (TGF-b and IL-10) by the UVB-induced Treg cells in mice. Mice (*XPA*-KO and their WT counterparts) which were provided a control diet or GSPs-supplemented diet were UVB irradiated and sensitized with DNFB. The mice were sacrificed 5 d after sensitization and Treg cells were positively selected from the single-cell suspensions prepared from the spleens and draining lymph nodes using Treg-specific magnetic beads from Miltenyi. (**A**) The percentages of Treg cells were analyzed using FACS analysis. (**B**, **C**) To examine cytokine production by Treg cells from WT (B) and XPA-KO (C) mice, equal numbers of Treg cells (2 × 10^6^) from mice were stimulated as detailed in Materials and Methods. The cell culture supernatants were collected 48 h later and the concentrations of TGF-b and IL-10 were measured using cytokine-specific ELISA kits. Significant inhibition versus UVB-treated control, *P < 0.001 (*n* = 5/group).

To determine the effect of GSPs on the development of Treg cells, C3H/HeN mice fed a GSPs-supplemented diet or a control diet were exposed to UVB radiation and then sensitized with DNFB as described in the Materials and Methods. The numbers of Treg cells were estimated by sorting of CD4^+^ cells from the spleens and lymph nodes of the mice and FACS analysis of the expression of CD25, and Foxp3. As shown in Figure 1A (upper panel), administration of dietary GSPs decreases the Treg cell population from 21.7% in UVB-exposed group to 11.8% in GSPs+ UVB-irradiated wild-type mice. These results indicate that GSPs inhibit the development of immunosuppressive Treg cells in UVB exposed mice. As we have shown that GSPs inhibit UVB-induced immunosuppression by enhancing the repair of damaged DNA in UVB-exposed LC/DC of the skin [27], we further checked the effect of GSPs on the numbers of Treg cells in *XPA*-KO mice under identical experimental conditions. No significant differences were found in the numbers of Treg cells in UVB-irradiated *XPA*-deficient (*XPA*-KO) mice that were fed a diet supplemented with GSPs and UVB-irradiated *XPA*-KO mice that were fed the control diet (Figure 1A, lower panel).

To determine the effect of dietary GSPs on the functional activity of Treg cells in wild-type and XPA-deficient mice, the Treg cell population was sorted from lymph node and spleen preparations, placed in culture and the supernatants were collected. The levels of the immunosuppressive cytokines, IL-10 and TGF-β, in the culture supernatants were determined using cytokine-specific ELISA kits. As shown in Figure 1B, Treg cells from UVB-irradiated GSPs-fed wild-type mice produced significantly less IL-10 (65%, *P* < 0.001) and TGF-β (79%, *P* < 0.001) than Treg cells from UVB-irradiated wild-type fed the control diet. In contrast, dietary GSPs did not significantly inhibit the levels of IL-10 or TGF-β by Treg cells isolated from UVB-exposed *XPA*-KO mice (Figure 1C). These data suggest that dietary GSPs reduce the suppressive effects of UVB-induced Treg cells in mice, and further suggest that this GSPs-induced reduction in the production of immunosuppressive cytokines by Treg cells requires a functioning DNA repair mechanism.

### Dietary GSPs enhance the ability of Treg cells from wild-type mice, but not *XPA*-KO mice, to stimulate production of IFNγ by T cells

**Figure 2:**
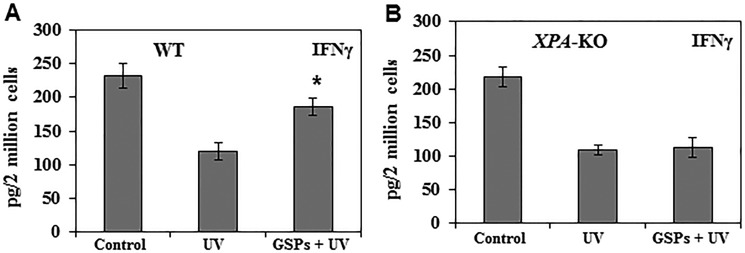
Dietary GSPs stimulate the production of IFNγ by UV-induced Treg cells (CD4+ CD25+ cells) in mice Treg cells were purified, as described in Materials and Methods, from the lymph nodes and spleens of mice (WT and *XPA*-KO) that were UVB irradiated and DNFB sensitized and were provided either the standard diet or the GSPs-supplemented diet. The Treg cells (2 × 10^6^) were then placed in culture with DNFB-primed CD8^+^ T cells that were isolated from naïve mice that had not been UVB-irradiated or fed GSPs. The cell culture supernatants were collected 48 h later and concentration of IFNγ was measured using an IFNγ-specific ELISA kit. (**A**) Data in wild-type mice. (**B**) Data in *XPA*-deficient mice. Significant increase versus UV alone exposed group of mice, *P < 0.001, *n* = 5/group.

To verify that dietary GSPs can inhibit the functions of Treg cells from UVB-irradiated mice and that this can contribute to the prevention of UVB-induced immunosuppression, we tested whether Treg cells from GSPs-treated mice can stimulate the production of IFNγ by CD8^+^ T cells. For this purpose, Treg cells were isolated from the spleens and lymph nodes of the mice and then co-cultured for 48 h with CD8^+^ T cells and bone marrow-derived dendritic cells from naïve mice that had not been UVB irradiated or fed GSPs. Cell culture supernatants were collected for the analysis of IFNγ by ELISA. The levels of IFNγ production were significantly lower (73%, *P* < 0.001) in the supernatants of co-cultures in which the Treg cells were obtained from UVB-irradiated wild-type mice than in the supernatants of co-cultures in which the Treg cells were obtained from wild-type mice that were not UVB-irradiated, confirming the immunosuppressive effects of Treg cells in UV-irradiated mice. The levels of IFNγ in the supernatants from the co-cultures in which the Treg cells were obtained from UVB-irradiated wild-type mice that had been fed GSPs were significantly higher (70%, *P* < 0.001) than in the co-cultures in which the Treg cells were obtained from UVB-irradiated wild-type mice that had not been fed GSPs (Figure 2A). In contrast, the levels of IFNγ were not significantly higher in the supernatants obtained from co-cultures in which the Treg cells were obtained from UVB-exposed *XPA*-KO mice that were fed GSPs than in the supernatants obtained from co-cultures in which the Treg cells were obtained from UVB-exposed *XPA*-KO mice not fed GSPs, as shown in Figure 2B. These data suggest that dietary GSPs inhibit the functional activity of Treg cells in UVB-irradiated wild-type mice as indicated by the greater secretion of IFNγ by CD8^+^ T cells on co-culture with the Treg cells. The absence of this effect of GSPs on Treg cells obtained from *XPA*-KO mice provides further evidence that the effects of GSPs on the function of Treg cells are associated with repair of UVB-induced DNA damage.

### GSPs prevent UVB-induced immunosuppression by decreasing the functional activation of Treg cells in UVB-irradiated mice: Evidence from adoptive transfer experiments using Treg cells

**Figure 3:**
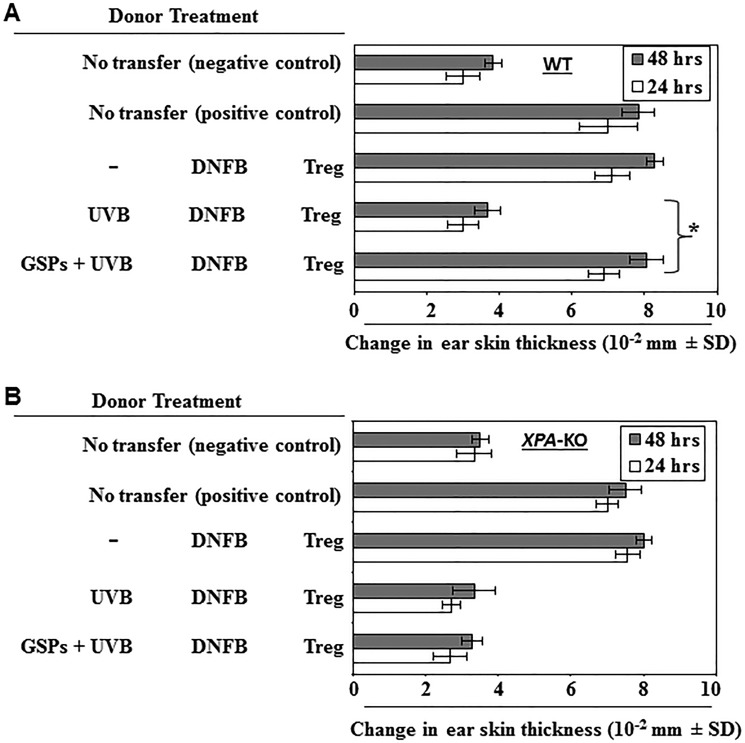
Effect of GSPs on adoptive transfer of immunity GSPs prevent transferable UVB-induced immunosuppression in wild-type (WT) mice through modulation of the activity of Treg cells, but do not inhibit UVB-induced suppression of the CHS response in *XPA*-deficient mice, which are unable to repair UVB-induced DNA damage. (**A**) Donor mice (WT counter-parts of *XPA*-KO mice) which were provided either the standard diet or the GSPs-supplemented diet were UVB-irradiated and sensitized, as detailed the in Materials and Methods. Mice were sacrificed 5 d after sensitization and Treg cells were positively selected and injected *i.v*. (1 × 10^6^) into naïve wild-type recipient mice which were DNFB sensitized 24 h after the adoptive transfer of cells. The recipient mice were challenged by application of DNFB to the ear 5 d after the sensitization and the change in ear skin thickness measured at 24 h and 48 h after the challenge. The change in ear skin thickness is reported as the mean of millimeters (10^−2^ mm) ±SD, *n* = 5 per group. Experiments were repeated once. Significant increase in CHS response *vs* UVB-irradiated control mice, *P < 0.001. (**B**) Experiments were conducted using *XPA*-KO mice under conditions identical to those described for Panel A. Dietary GSPs do not prevent transferable UVB-induced immunosuppression from *XPA*-KO mice to their naïve wild-type counterparts.

The above results suggest that dietary GSPs inhibit the UVB-induced activity of Treg cells, as indicated by suppression of IL-10 and TGF-β production by the Treg cells and an enhanced ability of the Treg cells to stimulate production of IFNγ by CD8^+^ T cells (Figures 1 and 2). We therefore carried out adoptive transfer experiments to verify the role of the effects of GSPs on Treg cells that could inhibit UVB-induced immunosuppression. As described in detail in the Materials and methods section, in these adoptive transfer experiments the wild-type donor mice were provided a standard diet or a standard diet supplemented with GSPs (0.5%, w/w), exposed to UVB, and sensitized to DNFB. The mice were sacrificed and the lymph nodes and spleens harvested 24 h after sensitization. Treg cells were purified from single-cell suspensions of the lymph nodes and spleens and then injected (1 × 10^6^) *i.v*. into naïve wild-type mice. The recipient mice were DNFB sensitized, challenged by application of DFNB to the ear skin 5 d later and the change in ear skin thickness measured at 24 h and 48 h after challenge. As shown in Figure 3A, the naïve mice that received Treg cells from UVB-exposed, wild-type donor mice that had been provided GSPs in their diet showed a significantly greater CHS response (*P* < 0.001) than the naïve mice that received Treg cells from the UVB-exposed wild-type mice that were not provided GSPs in the diet. Although the CHS response after challenge with DNFB was slightly greater at 48 h after challenge than 24 h after challenge, the difference was not statistically significant. These results indicate that the inhibition of UVB-induced suppression of CHS by dietary GSPs is mediated primarily through the functional inactivation of Treg cells. The same adoptive transfer protocol was carried out using cells from *XPA*-KO donors. The preventive effect of GSPs on the UVB-induced suppression of CHS was not seen in the naïve mice which had received *i.v*. Treg cells from UVB-exposed and GSPs-fed *XPA*-KO mice (Figure 3B). This observation suggests that the dietary GSPs-mediated functional inactivation of Treg cells in UVB-exposed mice is dependent on the effects of the GSPs on DNA repair in the skin cells.

**Figure 4:**
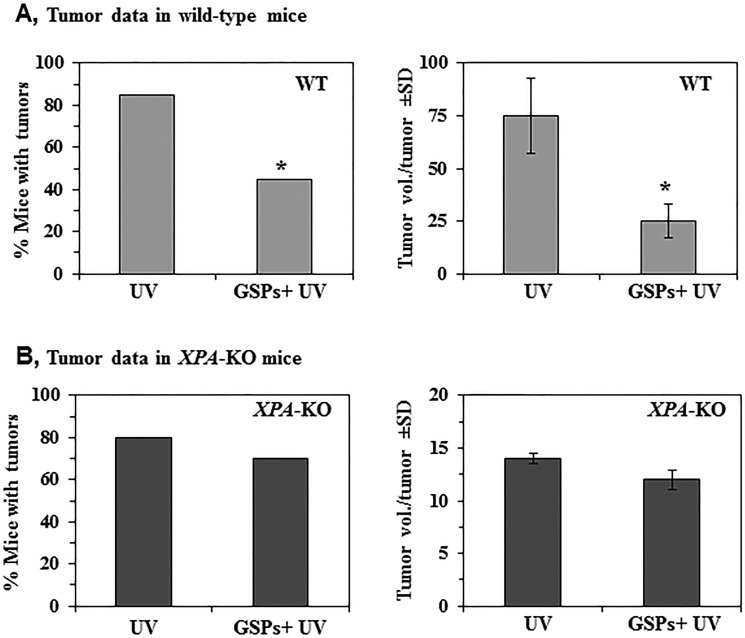
Effect of dietary GSPs on photocarcinogenesis *XPA*-KO and their WT counterparts were subjected to photocarcinogenesis protocol, as detailed in Materials and Methods. Mice were given either control AIN76A diet or GSPs-supplemented control diet (0.5%, w/w) throughout the experiment. (**A**) Dietary GSPs inhibit UVB-induced skin tumor development in WT mice in terms of tumor incidence and tumor growth or size. The resultant tumor data are presented at the termination of the experiment at 30^th^ week of the experiment. Significant inhibition versus control group of mice, *P < 0.001. (**B**) Dietary GSPs failed to significantly inhibit UVB-induced skin tumor development in *XPA*-KO mice, as is evident by the tumor data presented in terms of tumor incidence and tumor volume/tumor at the termination of the experiment at 30^th^ week. The tumor volume in each treatment group was recorded at the termination of the photocarcinogenesis experiment, and represented in mm^3^ as mean ± SD, *n* = 10 per group.

To determine whether the functional inactivation of Treg cells by GSPs results in inhibition of photocarcinogenesis in mice and whether the NER mechanism is involved in this process, we examined the effect of dietary GSPs on photocarcinogenesis in *XPA*-KO mice and resultant data were compared with the skin tumor data obtained from wild-type mice. Using a standard photocarcinogenesis protocol, as described in the Materials and Methods section, we have shown previously that dietary GSPs prevent photocarcinogenesis in C3H/HeN (wild-type) mice [28] compared with non-GSPs-treated C3H/HeN mice, and representative data from the previous study are reproduced here in Figure 4A. Dietary GSPs inhibited UVB-induced skin tumorigenesis in C3H/HeN mice as determined by tumor incidence (40%, *P* < 0.001) and growth (size) of the tumors (67%, *P* < 0.001). In contrast, dietary GSPs did not significantly inhibit UVB-induced skin tumor development in *XPA*-KO mice as compared to tumor development in *XPA*-KO mice that were not provided as GSPs-supplemented diet. Tumor development was evaluated in terms of percent of mice with tumors and tumor volume/tumor in these mice (Figure 4A and 4B) as determined at the termination of the photocarcinogenesis experiment (30th week).

### Dietary GSPs affect the levels of immunoregulatory cytokines in the tumor microenvironment of wild-type, but not *XPA*-deficient, mice

**Figure 5:**
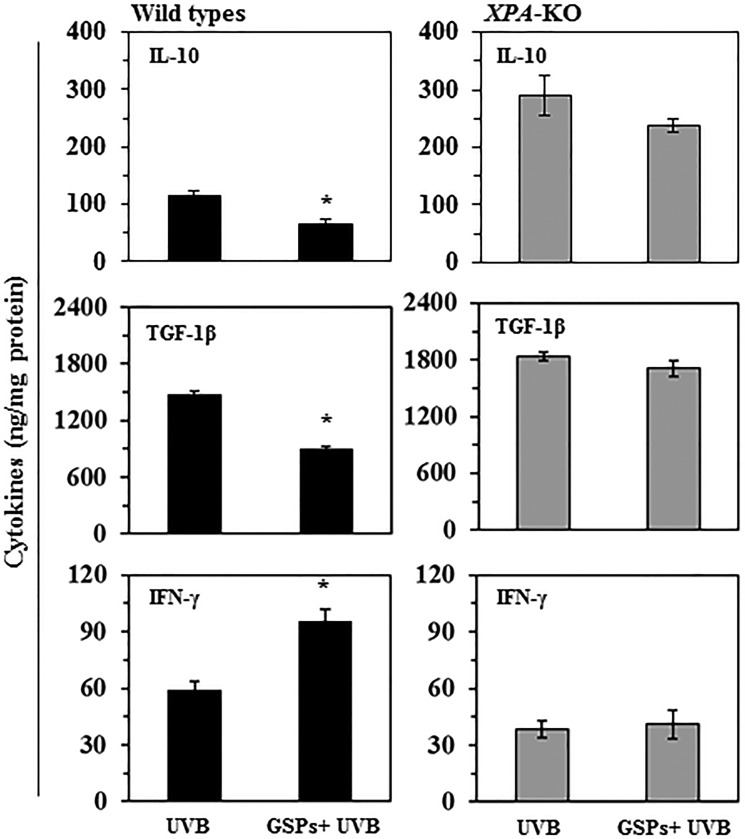
Effect of dietary GSPs on the levels of cytokines in UVB-induced skin tumors Effect of dietary GSPs on the levels of cytokines in UVB-induced skin tumors. At the end of photocarcinogenesis protocol, mice were euthanized and tumor tissues were collected from *XPA*-KO mice and their wild type counterparts. The levels of cytokines in tumor samples were determined using cytokine-specific ELISA following the manufacturer's protocol. Each sample was a pool of three tumor tissues obtained from different mice of the same group. Results of cytokines are presented as ng/mg protein as means ± S.D, *n* = 6. Statistical significant versus non-GSPs-treated UVB exposed control, *P < 0.001.

To determine the effects of GSPs on the immunoregulatory cytokines in the tumor microenvironment, homogenates of tumor tissues were analyzed for expression of IL-10 and TGF-1β as well as IFNγ using cytokine-specific ELISA kits. As shown in Figure 5, we did not find a significant difference in the levels of IL-10, TGF-1β or IFNγ in tumors from *XPA*-deficient mice that were provided GSPs in their diet and those that were not (right panels). In contrast, the levels of IL-10 and TGF-1β were significantly lower (*P* < 0.001) and the levels of IFNγ were significantly higher (62%, *P* < 0.001) in the skin tumors from GSPs fed, UVB-irradiated wild-type mice as compared with the levels of these cytokines in the skin tumors of UVB-irradiated wild-type mice that were not fed GSPs (left panels). These data indicate that GSPs have the ability to alter the tumor microenvironment and are thus able to block or slow the growth of UVB-induced skin tumors. They also provide further evidence that the chemopreventive actions of GSPs are mediated through repair of UVB-induced DNA damage medicated by NER.

## DISCUSSION

Nonmelanoma skin cancers, including basal cell carcinoma and squamous cell carcinoma, represent the most common malignant neoplasms in humans, particularly in Caucasians. Chronic exposure to UV radiation is a well-recognized etiologic factor for skin cancer risk, and UV-induced immunosuppression has been implicated in the risk of cutaneous malignancies. Although multiple mechanisms have been identified that may contribute to UV-induced immunosuppression, there is evidence that UV induction of Treg cells plays a central role in both UV-induced immunosuppression and initiation of skin carcinogenesis [9, 10]. Treg cells, including UV-induced Treg cells, act primarily to suppress the activation of T cells and immune responses [10, 29]. To develop effective strategies for the prevention of UVB-induced immunosuppression, we have assessed the effects of selected phytochemicals on UV-induced immunosuppression using *in vivo* mouse models. We have shown previously that dietary GSPs inhibit UVB-induced immunosuppression, as demonstrated by inhibition of UVB induced suppression of the CHS response to DNFB, by enhancing the repair of UVB-induced DNA damage and also by enhancing the functional activity of dendritic cells in the UVB-exposed mouse skin [27]. These dendritic cells migrate to the lymphatic system and play a role in T cell activation. As these are complex processes, GSPs could potentially be acting through multiple different molecular targets associated with diverse mechanistic pathways. Our current novel data suggest that dietary GSPs not only blocks the development of Treg cells in UVB-exposed mice, they also inhibit the functional activity of Treg cells as indicated by suppression of the ability of the Treg cells to promote production of immunosuppressive cytokines (IL-10 and TGF-β) and to inhibit IFNγ production by T cells.

As we had shown earlier that inhibition of UV-induced immunosuppression by GSPs is mediated through rapid repair of UVB-induced DNA damage, we further tested the effects of dietary GSPs on Treg cell development and Treg cell activity in *XPA*-KO mice, which lack NER capability. Dietary GSPs failed to inhibit the functional activity of Treg cells from *XPA*-KO mice as indicated by their lack of suppression of the ability of the Treg cells to promote production of immunosuppressive cytokines (IL-10 and TGF-β) and inhibit IFNγ production by T cells. Collectively, these new data indicate that dietary GSPs affect the production of immunosuppressive as well as immunostimulatory cytokines by a mechanism that involves the regulatory T-cell population. The data also suggest the ability of GSPs to restore the function of T cells in terms of the ability of these phytochemicals to suppress the functional ability of regulatory T-cells in UV-exposed mice.

As the hapten-specific effects of the GSPs on UV-induced immunosuppression can be adoptively transferred into naïve mice, we utilized an adoptive transfer approach to characterize the role of Treg cells (CD4+/CD25+ cells) in the GSPs-mediated effects in UV-exposed mice. For this purpose, we tested whether dietary GSPs inhibit the immunosuppressive activities of Treg cells in UV-exposed mice and whether GSPs stimulate the CHS response following adoptive transfer. Transfer of Treg cells from UVB-irradiated wild-type mice that had been fed GSPs to naïve mice resulted in a higher CHS response to DNFB than that observed in naïve mice that received Treg cells from UV-exposed wild-type mice that were not fed GSPs. Under identical conditions, adoptive transfer of Treg cells from UVB-irradiated *XPA*-KO mice that were fed GSPs to naïve mice did not induce a CHS response to the contact sensitizer, DNFB. This appears to be associated with the inability of GSPs to affect the functional activity of Treg cells obtained from *XPA*-KO mice. Further, the photo-immunoprotective effects of GSPs were verified in photocarcinogenesis experiments. Dietary GSPs do not have the ability to inhibit UVB-induced skin tumor development in *XPA*-deficient, *i.e*., NER-deficient mice, but have the ability to inhibit UVB-induced skin tumor development in their wild-type counterparts. The anti-photocarcinogenesis effect of GSPs in the wild-type mice was found to be associated with the reduced secretion of immunosuppressive cytokines and increased secretion of immunostimulatory cytokine IFNγ in the tumor microenvironment.

**Figure 6:**
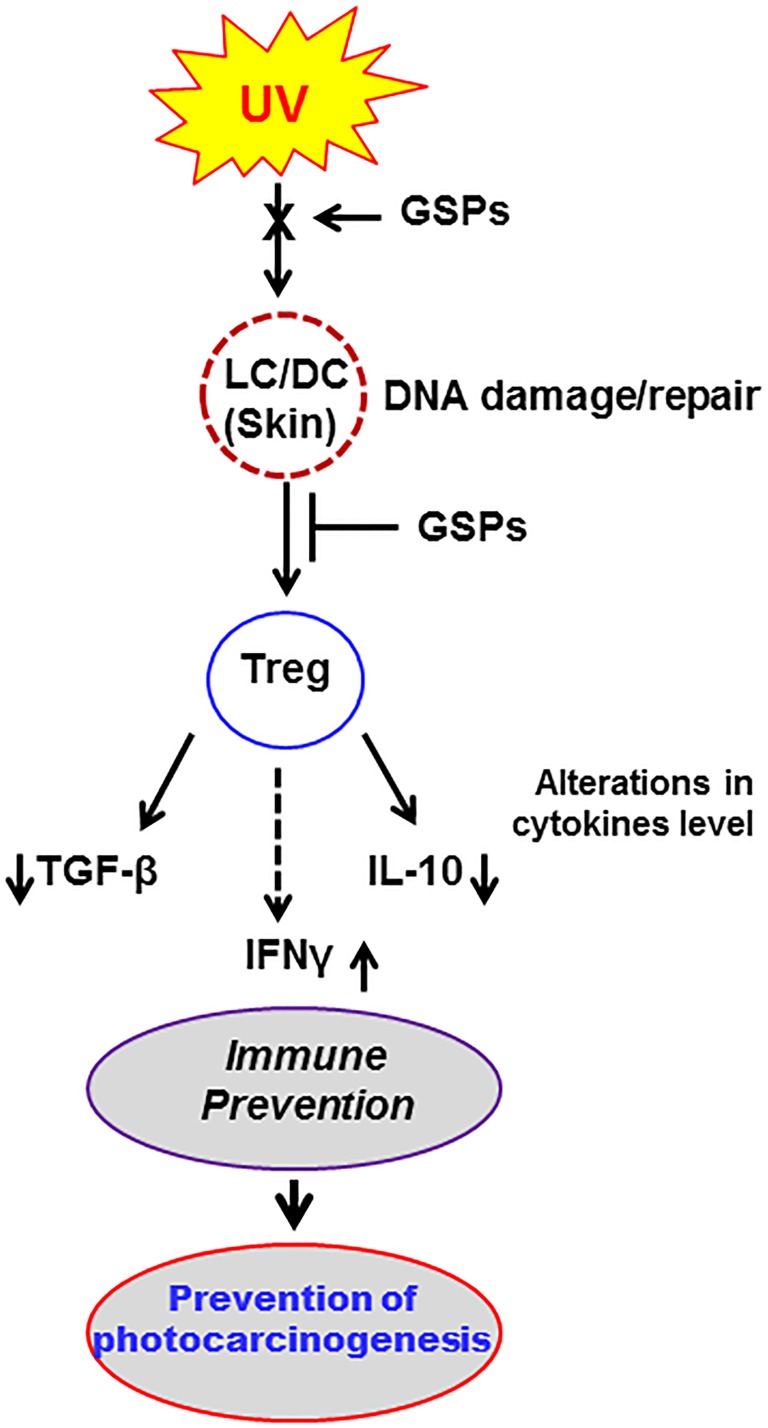
Summary of the effect of dietary GSPs in UVB-exposed mice Schematic diagram outlining a proposed model of the effects of dietary GSPs on UVB-induced immunosuppression and photocarcinogenesis in which inhibition of UV-induced immunosuppression by dietary GSPs is mediated through repair of UV-induced DNA damage in UV-exposed skin cells (LC/DC). This results in DNA repair-dependent functional inactivation of Treg cells in mice. Decreased production of immunosuppressive cytokines by Treg cells and simultaneously increased production of IFNγ leads to inhibition of UV-induced immunosuppression in mice fed GSPs.

Collectively, the results of this study suggest that the NER mechanism plays a central role in the photo-immunoprevention characteristics of GSPs. They suggest a model (Figure 6) in which the ability of GSPs to exert immunoprotective effects against UV radiation-induced suppression of immune system is mediated, at least in part, through functional inactivation of Treg cells in mice, and that this is associated with the ability of GSPs to repair damaged DNA in UVB-exposed skin through NER. This repair of damaged DNA in DCs helps the proper presentation of antigens to T cells in lymph nodes and that leads to the inhibition of Treg cell development. Although some phytochemicals, such as green tea polyphenols and silymarin, have been shown to protect against UVB radiation-induced immunosuppression [30, 31], the inactivation of the immunosuppressive function of Treg cells in UVB-exposed animals is a novel target for GSPs. These findings with GSPs are in line with the reports that suggest that the susceptibility to UVB radiation is increased in mice lacking NER mechanism [32]. These results also demonstrate that the photo-immunoprotective effect of dietary GSPs can be used as an alternative strategy to stimulate the immune system and that can help to protect against non-melanoma skin cancers in high risk individuals.

## MATERIALS AND METHODS

### Animals

The *XPA*-KO mice on a C3H/HeN background were bred in our Animal Resource Facility, as described previously [30, 31]. Female C3H/HeN mice of 4 to 6 weeks of age were purchased from Charles River Laboratories. All mice were maintained under standard conditions (12-hour dark/12-hour light cycle) with a temperature of 24°C ± 2°C and relative humidity of 50% ± 10%. The mice were provided a control AIN76A diet with or without supplementation with GSPs and drinking water *ad libitum* throughout the experiment. Mice in the GSPs-fed group were provided the GSPs-containing diet from 7 days before the start of UV irradiation until the end of the experiment. The animal protocol used in this study was approved by the Institutional Animal Care and Use Committee of the University of Alabama at Birmingham, Birmingham, AL.

### Chemicals, antibodies, and GSPs

The CD4^+^CD25^+^ Regulatory T cell Isolation Kit and CD8^+^ T cell Isolation Kit were purchased from Miltenyi Biotec (Auburn, CA). Anti-mouse CD45R/B220 antibody used for preparation of bone marrow-derived dendritic cells was purchased from BD Bioscience (San Diego, CA), while antibodies directed against CD4 (GK1.5), CD8 (Lyt-2), and HB-32 were a kind gift from Dr. Xu of the University of Alabama at Birmingham. Dynabeads coupled with anti-rat IgG antibodies were purchased from Invitrogen (Carlsbad, CA). IL-2, IL-4, DNFB and lipopolysaccharide (LPS) were purchased from Sigma Chemical Co. (St. Louis, MO). Anti-mouse CD3e, anti-mouse CD28 and GM-CSF were purchased from BD Bioscience (San Diego, CA). Mouse-specific ELISA kits for TGF-β, IL-10, and IFNγ were purchased from eBioscience (San Diego, CA). The GSPs were obtained from the Kikkoman Corporation (Japan) and the chemical composition of this product has been described previously [25, 26]. Experimental diet containing GSPs (0.5%, w/w) was prepared commercially in pellet form in the AIN76A-powdered control diet by TestDiet (Richmond, IN) using the GSPs that we provided.

### UVB irradiation

The clipper-shaved backs of the mice were UVB-irradiated using a band of 4 FS20 UVB lamps (Daavlin; UVA/UVB Research Irradiation Unit, Bryan, OH) equipped with an electronic controller to regulate UV dosage, as described earlier [26, 27]. The UV lamps emit UVB (280–320 nm; ∼80% of total energy) and UVA (320–375 nm; 20% of total energy), with UVC emission being insignificant. We used two different doses of UVB irradiation depending on the nucleotide excision repair (NER) capability of the mice used in this study. *XPA*-KO mice lack DNA repair genes or NER genes and are sensitive to UVB radiation-induced DNA damage. For this reason, 20 mJ/cm^2^ dose of UVB was used for irradiation of *XPA*-KO mice. In the case of C3H/HeN mice (wild-type counterparts of *XPA*-KO mice), a dose of 150 mJ/cm^2^ UVB irradiation was used.

### Purification of CD4^+^CD25^+^ Treg cells

Treg cells were isolated from the draining lymph nodes and spleens of mice using the CD4^+^CD25^+^ Regulatory T cell Isolation Kit purchased from Miltenyi Biotec according to the instructions provided by the manufacturer. Briefly, the procedure involves pre-enrichment of CD4^+^ T cells by depletion of non-CD4^+^ T cells by magnetic labeling using a cocktail of biotin-conjugated antibodies (antibodies directed against CD8a, CD11b, CD45R, CD49b and Ter-119) and anti-biotin microbeads. In parallel, the cells are labeled with CD25-PE. The cell suspension is first loaded on to a MACS Column placed in the magnetic field of a MACS separator. The non-CD4^+^ T cells, which are magnetically labeled, are retained on the column. The CD4^+^ T cells, which are not retained on this column, are collected, magnetically labeled with anti-PE microbeads and the CD4^+^CD25^+^ are positively selected using the magnetic separator.

### Preparation of bone marrow-derived dendritic cells (BM-DCs)

BM-DCs were prepared from bone marrow as described previously [27]. Normal C3H/HeN mice were sacrificed and the femurs were collected, cleaned and then sterilized by dipping in 70% ethanol for 5 min. The bone marrow cells were collected in RPMI 1640 media under a sterile hood. After lysis of red blood cells using ammonium, chloride, potassium (ACK) cell lysis buffer, the B cells and T cells were depleted using antibodies against CD45R/B220, CD4 (GK1.5), CD8 (Lyt-2), and HB-32 and Dynabeads. The remaining cells were washed, suspended in dendritic cell medium [RPMI supplemented with 10% FBS, GM-CSF (10 ng/ml) and IL-4 (10 ng/ml)], and cultured in this media for 5 d. LPS (5 μg/ml) was then added to the culture media to induce maturation of dendritic cells and the cells harvested the following day. These BM-DCs were ≈95% CD11c^+^ cells.

### Purification of CD8^+^ T-cell subpopulations

Purification of CD8^+^ T cells from single-cell suspensions of the spleens and lymph nodes of the sensitized mice and naïve mice was carried out using rat anti-mouse CD8 monoclonal antibody and the MACS system following the manufacturer's instructions (Miltenyi Biotech, Inc.). The efficiency of positive-selection of T-cell subpopulations was examined by flow cytometry (EPICS XL, Coulter, Miami, FL) using specific antibodies to target cells.

### Analysis of IFNγ secretion by T cells under the influence of Treg cells

Mice were UVB irradiated with and without treatment of dietary GSPs as described above and sensitized by painting DNFB (25 μl of 0.5%) on the UVB-irradiated skin site 24 h after the last UVB exposure. The mice were sacrificed 5 d later, the spleens and draining lymph nodes collected, single-cell suspensions prepared and CD4^+^CD25^+^ Treg cell subpopulations were purified as described above. Purified CD4^+^CD25^+^ Treg cells (2 × 10^6^) prepared from different treatment groups were then placed in culture with CD8^+^ T cells (2 × 10^6^) and DNBS-labeled BM-DCs (2 × 10^5^) for 48 h. Both CD8^+^ T cells and BM-DCs were prepared from wild-type mice that were not provided GSPs in their diet and were not UVB irradiated. After 48 h of co-culture, the cell culture supernatants were collected by centrifugation for the analysis of IFNγ using an ELISA kit.

### Analysis of TGF-β and IL-10 secretion by Treg cells

Mice (*XPA*-KO and their WT counterparts) were UVB irradiated for four consecutive days with and without GSPs treatment and sensitized with DNFB 24 h after the last UV exposure as described above. The mice were sacrificed 5 d later and the Treg cells positively selected from the single-cell suspensions prepared from spleen and lymph nodes using Treg-specific magnetic beads from Miltenyi. To examine cytokine production, equal numbers of Treg cells (2 × 10^6^) were stimulated with anti-CD3 (5 μg/ml) and CD28 (10 μg/ml) in presence of IL-2 (20 ng/ml). The cell culture supernatants were harvested 48 h later and the concentrations of TGF-b and IL-10 were measured by ELISA.

### Adoptive transfer of Treg cells and assessment of CHS response

For adoptive transfer of CD4^+^CD25^+^ Treg cells, the donor mice were exposed to UVB radiation (150 mJ/cm^2^; *XPA*-KO, 20 mJ/cm^2^) for four consecutive days. The mice were sensitized to DNFB 24 h after the last UVB exposure as described above. Five days after sensitization, they were sacrificed, the draining lymph nodes and spleens were harvested and single-cell suspensions prepared. CD4^+^CD25^+^ Treg cells were purified as described above and injected *i.v*. (1 × 10^6^ CD4^+^CD25^+^ Treg cells/mouse) into untreated naïve C3H/HeN mice. The recipient mice were sensitized by the epicutaneous application of DNFB on the shaved abdominal skin and challenged with DNFB on the ear skin 5 d after sensitization. The ear swelling response was determined by measuring the ear skin thickness at 24 h before and 24 and 48 h after the challenge. Groups of naïve mice, which were not sensitized but were ear challenged, served as a negative control.

### Photocarcinogenesis protocol

The *XPA*-KO mice and their wild-type counterparts (C3H/HeN mice) were divided into three treatment groups with 10 mice in each group. These groups of mice included a: (*i*) Control group (not UVB-irradiated and not fed a GSPs-supplemented diet); (*ii*) The UVB-irradiated control group (mice that were exposed to UVB but not fed a GSPs-supplemented diet); and (*iii*) The GSPs+ UVB group (mice that provided a GSPs-supplemented diet (0.5%, w/w) from 7 d prior to UVB irradiation until the termination of the photocarcinogenesis experiment). The photocarcinogenesis protocol used has been described previously [28]. Briefly, the shaved backs of the mice were irradiated with UVB (wild-type, 200 mJ/cm^2^; *XPA*-KO, 20 mJ/cm^2^) three times per week for a total of 30 weeks. The backs of the mice were shaved again using clippers if hairs grew on the skin during the photocarcinogenesis experiment, and examined on a weekly basis to check for the growth of papillomas or tumors. At the termination of the experiment, the dimensions of all the tumors on each mouse were recorded. Tumor volumes were calculated using the hemiellipsoid model formula: tumor volume = 1/2 (4π/3) (*l*/2) (*w*/2) *h*, where *l* = length, *w* = width and *h* = height.

### Statistical analysis

The differences between experimental and control groups in terms of the CHS response and the levels of cytokines were analyzed using the Student's *t* test and using one-way analysis of variance (ANOVA) using GraphPad Prism version 4.00 for Windows, GraphPad Software, (San Diego, CA) USA. In each case *P* < 0.05 was considered statistically significant.
